# Supplementary material for: Are carbon emissions trading and green financial instruments synergistic? -Comprehensive quantitative research based on content analysis
Source: PLoS One. 2024 Mar 7;19(3):e0298601. doi: 10.1371/journal.pone.0298601 (PMC10919601; doi:10.1371/journal.pone.0298601)
Supplement: S1 Data — (ZIP) [file pone.0298601.s001.zip › Data/地级市Z_F工作报告.zip!/║■▒▒/╗╞╩»╩╨/└┤╫╘╣1⁄2╓┌║┼í╛┬φ┐╦╩2╛▌═°í┐_2018.docx]

各位代表：

现在，我代表市人民政府向大会报告工作，请予审议，并请市政协各位委员和列席人员提出意见。

一、2017年工作回顾

过去一年，是党的十九大胜利召开之年，也是新一届政府的开局之年。一年来，在市委的坚强领导和市人大、市政协的监督支持下，我们始终坚持稳中求进的工作总基调，坚持以供给侧结构性改革为主线，大力实施工业强市赶超发展战略，狠抓项目攻坚行动，顶住经济下行压力,保持转型发展定力，增强爬坡过坎耐力，经济发展跨过谷底、实现反转，初步走出了一条经济平稳增长与结构优化升级相互促进、新旧动能有效接续转换的创新转型之路。

——主要经济指标实现难中求进、稳中向好。初步预计，地区生产总值增长7.2%，规模以上工业增加值增长8%，固定资产投资增长12%，地方公共财政预算收入增长5.2%，外贸出口增长17%，社会消费品零售总额增长11%，城乡居民人均可支配收入分别增长8.6%、8.2%，城镇新增就业5.2万人,全面完成省定节能减排任务。随着转型的加速，今后一个时期将会有一个更好的预期。

——工业强市战略成效初显。工业稳住全市发展大局，对经济发展的贡献率超过60%，规上工业利润增幅、产品产销率居全省前列。工业技改投资翻番，传统产业“老树发新芽”，8家企业入选全省百强，数量全省第二，在全国占有相当份额的模具钢产业抱团重组、提档升级，重新焕发生机。欣兴电子、定颖电子等项目建成投产，黄石已成为国内第三大PCB（1）产业聚集区，第一辆“黄石造”汉龙汽车整车下线、实现量产，多年蓄积的产业新动能开始释放。成功获批全省唯一一家国家产业转型升级示范区。跻身全国创新驱动助力工程示范市。创建全国质量强市示范城市通过预验收。大冶市全国百强进至79位。

——城市软实力有力彰显。成功举办中国（黄石）地矿科普大会、第二届全国工业旅游创新大会，“四大工业遗址”（2）串珠成线，大冶铁厂（新冶钢）入选第一批国家工业遗产，矿山公园获批国家工业遗址旅游基地，充分展示了“山水园林市、最美工业城”的独特魅力。黄石信用状况监测排名居全国前列。成功创建国家卫生城市，圆了全市人民19年的创卫梦。社会文明程度明显提升，大冶市跻身全国文明城市，新增3个全国文明镇村。

——发展平台更加坚实。大冶湖新区新开工重大产业项目22个、总投资236亿元，引爆发展的态势正在形成。黄石新港成功获批全省继武汉之后第二个全国多式联运（3）示范项目，开通韩国釜山等江海直航班线，建成长江中上游第一条公铁水无缝衔接多式联运线路。国家级黄石大冶湖高新区申报进入最后审批环节。武九客专全面运营，黄石正式并入国家高铁干线网，成功开通直达北京的高铁列车。黄阳一级路建成通车，形成市域半小时交通圈，百万阳新人民多年的夙愿今朝实现。

——改革动能持续释放。供给侧结构性改革深入推进，在实现全域无落后钢铁产能、无煤炭生产企业的基础上，又实现烟花爆竹生产企业全部退出，商品房库存去化周期保持在合理区间，黄石连续13年被评为全省金融信用市，为企业减负超过10亿元。“放管服”（4）改革深入推进，在全省率先推行“先建后验”（5）、“二十九证合一”（6）、“一个号码管服务”（7）等举措。内贸流通体制改革、物流标准化试点经验在全国复制推广。

——人民群众获得感明显增强。民生支出占财政全部支出80%以上，预算安排5.6亿元弥补企业职工养老保险缺口，力度前所未有。城镇居民医保与新农合并轨，惠及人口185万人。精准脱贫1.9万人，出列18个村。完成棚户区改造1.64万户，发放安置资金50多亿元。城市颜值品质进一步提升，生态产品供给更加充分。“一感两度两率”（8）位居全省第一方阵。

一年来，我们主要做了以下工作：

（一）坚持工业引领，推进产业转型

全力振兴黄石制造。以国家产业转型升级示范区建设为抓手，突出产业提升价值链、产品迈向中高端，大力实施工业强市三年攻坚计划，实施“百企技改”工程，发放工业转型发展奖补资金1.2亿元，完成工业投资607亿元，其中技改完成220亿元，分别增长15.2%、118.6%，新增规上工业企业77家，黑色金属、有色金属、建材等传统产业增长均在20%以上，电子信息、新能源汽车、高端装备制造等新兴产业不断壮大，工业发展呈现产销两旺、质效提升的可喜局面。全面加强质量管理，新增中国地理标志产品（9）1个、湖北名牌产品7个。

着力服务实体经济。扎实开展第三轮“双千”（10）活动，财政预算安排企业人才引进、用工补贴等资金6800万元，帮助企业招工1.7万人，帮助360家企业解决银行续贷近30亿元，为企业解决各类难题800多个。

突破性发展现代服务业。坚持以工业旅游为引领，统筹推进乡村游、红色游、全域游，全市旅游总收入实现141.6亿元，增长20%。加快发展生产性服务业，新增3A以上物流企业11家，总数达到16家，全省排名第四。

加快发展现代农业。大力实施“一镇一业”工程，着力打造畜禽、水产、食品饮料三大产业链，组建油茶、稻虾共作、涉农电商三大产业联盟，新增省级农业产业化龙头企业3家、规上农产品加工企业6家，荣获省级农产品金牌6个。大冶被评为全国休闲农业和乡村旅游示范市。阳新被评为国家农村电商示范县和水生蔬菜全国优势特色区。全面完成农村土地确权任务。

（二）坚持项目支撑，扩大有效投资

强力推进精准招商。创新招商体制，狠抓产业链招商，成功引进海盈新能源、爱一城传感器等一批20亿元以上项目，新注册重点项目150个、增长32.7%，已开工125个、增长92.3%，实际到位内资1150亿元、增长28.6%，实际利用外资1.57亿美元、增长8%。

强力推进项目攻坚。对市级重点项目实行领导包保、现场拉练和挂牌督办，共实施亿元以上重大项目430个。汉龙汽车发动机、中铝铜箔等270个项目加快推进，中兴新先进材料一期、大冶有色30万吨阳极铜、宝钢产业园一期等160个项目建成投产。2个项目入选国家和省PPP（11）示范项目，8个PPP项目落地实施。

（三）坚持创新驱动，增强发展活力

增强发展新动能。新增高新技术企业37家，高新技术产业增加值增长12%。新增省级工程技术研究中心3家，组建黄石智能物流输送装备及工业机器人研究院，引进工信部电子第五研究所共建中国赛宝黄石工业技术研究院。完成重大科技成果转化65项，专利授权量1240件。

释放改革新动能。出台“金融八条”（12），6家平台公司资产规模超1100亿元，全年融资165亿元。成功发行2支企业债券，募集资金25亿元。新增省级银种子企业（13）20家，四板企业149家。出台人才“黄金十条”（14），新引进一批高层次人才及“产业工匠”在黄创业就业。积极推进大冶有色等31家国有企业“三供一业”（15）改革。“智慧政务”平台上线运行。

扩大开放新动能。引导企业参与“一带一路”建设，新增境外投资企业1家，新增境外投资5400万美元，华新水泥塔吉克斯坦和柬埔寨项目、橙石电子新加坡项目入选“一带一路”省重点支持项目库。复制推广自贸区“贸易便利化”经验，新增外贸出口企业31家，完成外贸进出口总额32亿美元、增长32%，总量全省第二。

（四）坚持设施先行，推进市域一体发展

突出新区带动。大冶湖新区基础设施投资180亿元，四连山工业平台完成“九通一平”（16），城市综合馆基本完工，市民之家主体结构封顶，湖北地质馆建成开放，大冶湖通江口裁弯取直完成主体工程建设，奥体中心、鄂东医疗中心、社会福利中心、地下综合管廊等重点项目加速推进。

突出港城联动。完成黄石港口总体规划和黄石新港物流（工业）园扩园规划修编，总投资45亿元的新港现代物流园项目进展顺利，新港已建成5000吨级泊位9个，年吞吐能力达4000万吨，成为集散货、件杂、集装箱和多式联运业务功能于一体的现代化综合港区。

突出路网建设。完成交通投资104亿元。棋盘洲、武穴长江大桥及连接线、沿江大道新港段、团城山综合客运枢纽、大棋路改造等重点项目进展顺利，武汉至南昌高速黄石段、有轨电车等项目前期工作加紧推进。建成农村公路370公里，完成公路生命安全防护工程（17）1200公里，兴富公路、锦冶公路建成通车。

突出旧城提质。实施老城区基础设施提升改造及城市整理三年行动计划，开工城市整理项目30个，完成磁湖东路、青龙山路、湖滨二巷、马鞍山路等次干道和微循环改造，磁湖湿地公园一期建成开放。实施黄棉、陈家湾、李家坊等重点棚改项目15个、9034套（户）。实施城管执法体制改革，扎实开展市容环境综合整治。

（五）坚持生态优先，改善人居环境

狠抓突出环境问题整治。完成中央环保督察反馈问题年度整改任务。实施20蒸吨以下燃煤锅炉整治，淘汰老旧车黄标车，更新112台新能源纯电动公交车，PM10、PM2.5分别下降3.5%、3.6%，空气优良天数比例提高2.3个百分点。编制完成土壤污染防治规划，实施东钢等地块的治理与修复。启动沿江危化品企业搬迁工作。查处环境违法案件230件。

全面推行河长制。坚持把长江大保护作为压倒性任务，建立市县乡村四级河长制（18）体系，实现全市每一片水域有人管、有人查、全覆盖。投资9.5亿元，完成各类水利工程2600处。开工建设污水管网项目22个、建成80公里，完成河西、花湖、青山湖、磁湖4座污水处理厂提标升级，乡镇污水处理厂全面开工建设。长江黄石饮用水源地水质稳定达标，国控、省控考核断面达标率均为100%，磁湖、大冶湖、保安湖水质有效改善。

加强生态建设。“绿满黄石”行动深入推进，新增改造城市绿地60公顷,完成造林绿化16.6万亩。积极创建国土资源节约集约示范市，建成高标准农田24万亩，实施占补平衡项目35个，新增耕地6694亩。狠抓矿山地质环境综合整治，铜绿山铜铁矿等8家矿山被评为国家级绿色矿山。阳新县太平村入选全国改善农村人居环境示范村。

（六）坚持共建共享，增进民生福祉

扎实抓好就业和社会保障。坚持以“双创”促进就业，城镇登记失业率2.95%。城乡居民和退休职工养老金、城乡低保和农村五保供养再次提标，社会救助水平稳居全省第一方阵。全面落实困难残疾人生活补贴和重度残疾人护理补贴制度。机关事业单位养老保险改革全面实施，“五项保险”(19)新增扩面65万人（次）。

狠抓精准扶贫。聚焦“两不愁三保障”，（20）组建800支扶贫工作队驻村包保、结对帮扶。大力实施光伏扶贫，着力打造一批特色产业扶贫基地。财政列支扶贫资金1亿元，发放小额扶贫贷款3.2亿元，在阳新县探索建立了村级集体经济组织和农民适当分享矿业开发收益机制。贫困人口居民医保参保率达100%，资助贫困学生3.4万名，改造贫困户危房1530户，完成易地扶贫搬迁1378户。

协调发展社会事业。新增幼儿学位1.2万个，完成167所学校“改薄”（21）任务，阳新县、市开发区义务教育均衡发展工作通过国评，3所高中异地重建和改扩建，总投资13亿元的阳新教育城投入使用。全面取消公立医院药品加成，启动“一村一名大学生村医”计划,城区紧密型医共（联）体（22）基本建成。启动国家历史文化名城创建，加快推进现代公共文化服务体系建设，“三馆一站”（23）、广播电视覆盖率均达100%。全力筹办省第十五届运动会，统筹推进总投资52亿元的37个场馆及配套工程建设。成功举办省第十四届中学生运动会、市第十届运动会。

创新社会治理。扎实推进立体治安防控体系建设，着力织密“九张网”（24），突出抓好“严打”整治，西塞山区夺取全国综治最高奖“长安杯”。全国社区治理和服务创新实验区顺利通过验收。着力打造阳光、责任、法治、亲民信访，群众诉求渠道进一步畅通。实施安全生产监管领域改革，安全生产形势保持总体平稳。大力开展餐桌污染治理，食品药品监管基础进一步夯实。深入推进“七五”普法，市民法治意识不断增强。启动新一轮全国“双拥”模范城创建，军政军民关系更加和谐。

狠抓政府自身建设，把全面从严治党要求贯穿政府工作始终，“两学一做”学习教育常态化制度化扎实推进，政府系统党的建设全面加强。认真贯彻落实中央八项规定精神，“四风”整治和廉洁政府建设成效明显。全面推进法治政府建设，进一步完善重大行政决策机制，认真执行市人大及其常委会各项决议决定，认真办理人大代表建议和政协委员提案。聚焦重点工作、重点项目，坚持挂牌督办、追责问责、狠抓落实。

与此同时，审计、统计、外事、侨务、民族、宗教、对台、保密、档案、妇女儿童、老龄、慈善、援藏、援疆、援利川、国家安全、广播电视、新闻出版、防震减灾、民防、水文、气象、史志等各项工作都取得新成绩。

各位代表，过去的一年，困难比预想的要多，结果比预期的要好。这是省委省政府正确领导、亲切关怀的结果，是市委统揽全局、科学决策的结果，是人大、政协有效监督、倾力支持的结果，是全市人民团结奋斗、顽强拼搏的结果。在此，我代表市人民政府，向全市人民，向给予政府工作大力支持监督的人大代表、政协委员，向民主党派、工商联、无党派人士，各人民团体，向驻黄解放军、武警官兵，向所有关心支持黄石建设发展的海内外人士，表示衷心的感谢!

我们也清醒地认识到，前进道路上仍然存在不少困难和挑战，主要是：经济还处于深度转型阶段，发展韧性、接续动能、创新能力有待加强，持续稳定增长的基础还不牢固；工业投资占比较低，民间投资乏力，直接影响发展后劲；实体经济仍然比较困难，一些企业停产半停产，中小微企业融资难融资贵、结构性用工难等问题较为突出；生态治理任务仍然十分繁重，加快发展与生态保护两难问题依然存在；如期全面建成小康社会存在短板，服务业增加值占比等指标差距较大，民生欠账较多；少数干部担当不够、作风不实、效率不高等问题依然存在。我们一定正视困难和差距，不掩盖、不回避、不懈怠，采取坚决措施加以解决。

二、2018年工作安排

2018年，是贯彻党的十九大精神的开局之年，是改革开放40周年，是决胜全面建成小康社会、实施“十三五”规划承上启下的关键一年。做好今年政府工作，意义特别重大。

政府工作的总体要求是：全面贯彻党的十九大精神，以习近平新时代中国特色社会主义思想为指导，坚持稳中求进工作总基调，坚持新发展理念，按照高质量发展的要求，以供给侧结构性改革为主线，围绕建设富强创新大城文明美丽幸福黄石，深入实施工业强市赶超发展战略，加速推进城市转型，扎实打好“三大攻坚战”（25），加快建设全面小康社会、全省重要区域性增长极。

经济社会发展的预期目标是：地区生产总值增长7.8%左右，在实际工作中争取更好结果；规模以上工业增加值增长8%；固定资产投资增长11%；社会消费品零售总额增长11%；外贸出口增长9%；地方公共财政预算收入增长8%；居民消费价格指数控制在3.5%左右，城镇登记失业率控制在4.5%以内，城乡居民收入与经济增长基本同步，单位生产总值能耗下降3.7%，完成省定主要污染物减排任务。

全面建成小康社会综合指数达到90%以上，其中今年补短板、强弱项的预期目标是：服务业增加值占GDP比重提高1个百分点，R&D经费投入强度达到1.8%，高新技术产业增加值占GDP比重达到15%，文化产业增加值占GDP比重达到2.3%，农村贫困人口脱贫2.6万人。

全面贯彻党的十九大精神，实现上述目标要求，我们将着重把握以下四点。

一是着眼于建设现代化经济体系，更加突出解决好质量和效率问题。总量不大、质量不高、核心竞争力不强，是黄石经济发展的最大问题。面对新时代新要求，必须更加准确把握我国经济已由高速增长阶段转向高质量发展阶段的基本判断，更加清醒认识到我市经济发展正处在滚石上坡、深度转型的历史方位，更加突出质量第一、效益优先，更加聚焦工业、聚焦产业，加快建设国家产业转型升级示范区，全力推动质量变革、效率变革、动力变革，持续用力，久久为功，只要路走对了，就不怕遥远，坚定朝着品质兼优、实力更强的现代化经济体系迈开步、不停步。

二是着眼于建设现代化区域性中心城市，更加突出解决好实力和魅力问题。建设长江中游城市群区域性中心城市、打造全省重要区域性增长极，是复兴黄石、建设现代化强市的重要基础和条件。面对新时代新要求，必须坚持工业强市赶超发展，着力振兴黄石制造，加速产业提升价值链、产品迈向中高端，形成产业核心竞争力；必须坚持港城联动、以港兴市，加快建设“亿吨大港、百万标箱”的现代化港区，提升区域辐射服务功能；必须坚定不移走“工业+”创新发展之路，创建国家历史文化名城，彰显区域文化特色，打造最美工业城市，把工业文明转化为转型发展的不竭动力；二号首长必须厚植绿水青山就是金山银山的理念，突出治理“山水气土”，着力打造秀美宜居的滨水城市。以承办省第十五届运动会为契机，加快体育强市建设，打造时尚运动新城，满足人民对美好生活的新需求。

三是着眼于全面深化改革，更加突出解决好动力和活力问题。创新不够、动力不强、韧性不足，是当前黄石发展的最大短板。面对新时代新要求，必须以更大的力度全面深化改革，把发展的基点建立在创新驱动之上，找出并填平在以往发展阶段被掩盖、被忽视的各种低效率洼地，持续增强产业新动能，释放改革新活力，扩大开放新动力，全面提高全要素生产率，让各种要素资源有效汇聚黄石，成为转型发展的强大推力。

四是着眼于决胜全面建成小康社会，更加突出解决好不平衡和不充分问题。如期全面建成小康社会是党和政府作出的庄严承诺，既是民生问题、发展问题，更是政治问题。面对新时代新要求，必须紧扣我国社会主要矛盾变化的基本特征，坚持以人民为中心的发展思想，瞄准全面建成小康社会的重点、短板和弱项，突出打好“三大攻坚战”，统筹抓好中心城区与县（市）区、新区与老城区、传统产业与新兴产业、三次产业之间的协调发展，努力增强发展的民生底色，让人民群众生活得更加幸福、更有获得感！

今年，我们将重点抓好以下九个方面工作。

（一）着力抓好以先进制造业为根本的工业经济

改造提升传统产业。以有色金属、黑色金属、建材产业为重点，持续推进“百企技改”工程，加快大冶有色40万吨电解铜、新冶钢“三大”技改升级工程（26）、华新百年复兴基地（27）等重大项目建设，完成技改投入150亿元以上。深入开展“双千”活动，工业转型专项资金向技改和小进规企业倾斜，力争新进规企业80家以上。支持优势企业实施兼并重组，着力盘活潜力型困难企业。

培育壮大新兴产业。加快推进永兴隆电路板、特创电子产业园等项目建设，拉长PCB产业链条，推动电子信息产业向手机制造、显示面板、芯片制造等领域延伸拓展。加快实施汉龙汽车配套产业园、海盈新能源等项目，健全新能源汽车产业链条。加快智能小家电特色园区建设，推动家电产业终端化、智能化发展。大力推进中兴新先进材料二期、博天环保产业园等项目建设。

做大做强优势产业。大力推进智能输送装备产业园、精密制造、大型成套装备、工业机器人等项目建设，加快构建绿色高端装备制造体系。推进模具材料、模具制造产业园建设，支持模具钢企业转型升级，再造产业竞争新优势。加快推进劲牌白酒基地等项目建设，推动食品饮料产业特色化发展。加快远大医药园等项目实施，推动生物医药产业规模化发展。以美尔雅、美岛为龙头，推进纺织服装产业品牌化发展。

突出质量引领和创新驱动。深入实施增品种、提品质、创品牌工程，新增湖北名牌产品5个以上，成功创建全国质量强市示范城市。强力推进创新型城市建设，培育一批细分行业隐形冠军企业，新增高新技术企业50家以上。加强知识产权开发、保护和利用，推动科技成果转化60项以上。组建铜加工、模具钢、节能环保等产业研究院。推进物联网、云计算、大数据等体系建设，推动数字经济与实体经济、信息化与工业化深度融合。大力实施技能强市工程，培育更多创造型、适用型“产业工匠”。推进黄石人力资源服务产业园建设，引进一批高层次急需人才。激发和保护企业家精神，鼓励更多市场主体投身创新创业。

（二）着力抓好以生产性服务业为重点的现代服务业发展

发展现代物流业。加快棋盘洲现代物流园、传化公路港物流园、花湖现代物流园、润信农产品物流园等重点项目建设。推进制造业企业“主辅分离”（28），发展第三方物流。支持企业建立互联网销售平台，实现线上线下融合发展。积极对接顺丰机场，启动通用机场建设，发展临空经济。

发展现代金融业。积极引进各类金融机构，着力打造磁湖西岸金融产业集聚区。大力发展科技金融、普惠金融、绿色金融等新业态，加快推进金安金融物流等项目建设，推动金融与经济深度融合。加快多层次资本市场建设，新增上市和挂牌企业60家以上，发行4支企业债券、募集资金55亿元以上，支持阳新利用国贫县企业上市“绿色通道”扩大融资。

推进全域旅游。实施加快旅游业改革发展三年行动计划，调整完善旅游管理和投融资体制，积极创建国家旅游标准化示范城市。坚持以工业旅游为主体，带动研学游、乡村游、康养游、红色游、全域游深度融合。做好“四大工业遗址”保护与开发，研究旅游火车长期运营措施。推进重点景区景点提档升级，加快特色小镇建设。认真筹办第二届中国（黄石）地矿科普大会，大力发展会展经济、节会经济。支持铁山区国家级全域旅游示范区建设。

提升商贸业态。积极参与武鄂黄都市圈核心功能区建设，深化内贸流通体制改革，引进大型商贸项目在团城山、大冶湖新区布局，加快商业示范街区建设，打造鄂东最具活力的消费中心。加快社区菜市场、超市、便利店、家政服务店等便民网点建设，打造“8分钟生活圈”。积极培育“互联网+”新业态，大力发展共享经济。

（三）着力抓好以招商引资为龙头的项目攻坚

坚持第一力度抓招商。完善招商体制机制，切实提高招商工作的精准度、有效性，力争新注册重点项目160个以上，到位内资、利用外资实现较大突破。突出主导产业、优势产业，引进一批建链、补链、强链项目。着力推进重资产招商，量身定制代建厂房和基础设施，吸引客商带轻资产“拎包生产”。加快推进科技招商，鼓励科研人员以知识产权投资入股，引进一批技术密集型企业。深化与央企、省企对接合作，力争引进重点项目15个以上。

持续开展项目攻坚。健全领导包保、挂点服务机制，实行项目清单化、责任化管理，开工建设绿创科技、高端工模具产业园等重点项目200个以上，确保中铝铜箔、天玑智谷等150个重点项目竣工投产，力争固定资产投资完成1700亿元。积极抢抓国家新时期高质量发展的机遇，争取一批产业转型、生态环保、城市建设和民生保障等领域重点项目。

补齐工业和民间投资短板。坚持把工业投资作为主攻方向，按照“四个一批”（29）的思路，加快实施策划储备的295个、总投资1900亿元转型升级重大项目，力争工业投资增长20%左右。鼓励民营企业参与国有企业改革、参股混合所有制企业，推出一批产业、能源、市政、交通等领域PPP项目，全面实施市场准入负面清单制度，落实同等待遇，引导民间资本加强合作、扩大投资。

（四）着力抓好以新港为引擎的开放平台建设

加快港口建设。开工建设新港化工码头、砂石中心等项目，加快推进富池综合码头、新冶钢山南铁路连接线等项目建设，开通“翻坝第二通道”铁水联运黄（石）蓉（成都）班列，开通并加密江海直达日韩国际班轮、长江内河固定班轮航线，确保新港多式联运示范项目全面运营。扎实推进宝钢新港产业园等项目建设。

加快园区建设。按照差异化、组团化、特色化思路，制定园区产业发展规划，引导投融资平台、社会资本参与园区建设。推进特色园区建设，抓好精特轴承、新冶钢汽车零部件、康源特钢等企业退城入园工作。大力发展县域经济，支持每个县（市）区、园区根据产业优势、资源禀赋、基础条件，重点发展2-3个主导产业，重点工业乡镇、工业小区打造1个主导产业。

加快交通枢纽建设。力争完成交通投资110亿元，重点推进棋盘洲和武穴两座长江大桥、鄂州至咸宁高速黄石段建设，开工建设武汉至南昌高速黄石段、黄石至武汉一级公路城区段、315河金省道，建成棋盘洲长江大桥连接线、316国道军垦至金山店段。加快实施有轨电车项目，团城山综合客运站建成运营。建成一级、二级公路60公里以上。

扩大对外开放。坚持引进来和走出去并重，把开放的大门打得更开更大，引导企业参与“一带一路”建设。加强与武汉联动发展，主动对接光谷科技创新走廊建设。加快融入长江经济带，积极承接沿海产业转移，深化与沿江港口合作。抓好国家级出口基地建设，扩大棋盘洲保税物流中心辐射范围，加强口岸通关能力建设，积极申报综保区，力争进出口货运量较大增长。

（五）着力抓好以乡村振兴为重点的“三农”工作

发展现代农业。坚持以农业供给侧结构性改革为核心，突出发展油茶、中药材、水产等特色产业，加快推进黄石国家级农业科技示范园、大冶现代农产品加工园建设。培育扶持农业产业化龙头企业，新增“三品一标”（30）农产品5个。深入实施“互联网+农业”工程，支持农产品电商基地发展，新增农村实用人才培训示范基地3个，发展新型农业经营主体30家以上。

建设美丽乡村。全力发展中心村、保护特色村、整治空心村。实施“厕所革命”三年行动计划。完善农村生活垃圾“集收运处”体系，健全农村环境卫生管理长效机制。推行农村污水生态化处理，推进城镇污水管网向周边农村延伸，乡镇污水处理厂全部建成。完成“四好”农村公路（31）200公里。完成高标准农田整治9万亩，改造中低产田12.3万亩，新增耕地3000亩，治理水土流失面积10平方公里。修复水毁灾损工程416处，加固堤防28公里，清淤沟渠35公里，除险加固水库8座，新增供水受益人口2.6万人。

深化农村改革。推进农村土地经营权抵押贷款试点，鼓励组建农村土地股份合作社，积极推广农业土地托管服务新模式，引导土地适度规模经营。加快农村宅基地管理改革，探索空置宅基地退出复垦利用模式。加快供销合作社及国有农场改革。深化农业水价改革。鼓励市民下乡、能人回乡、企业兴乡，发展民宿、农耕文创产业，建设田园综合体，推动农村资源与城市消费、农民增收与市民体验无缝对接。

（六）着力抓好以市域一体为格局的全域协调发展

加快新区建设。创新开发区体制机制。编制完成大冶湖生态城和黄石科教城总体规划，完成章山新材料产业园、太子临港产业园“九通一平”6000亩以上，建成投产重点产业项目10个以上。加快产城融合，实施邻里中心（32）、青年公寓、星级酒店等重点项目，建成城市综合馆、市民之家、社会福利中心、园博园二期等重点工程，完善新区配套功能。完成大棋路综合改造、奥体大道西延、大冶湖堤防加固等重点工程，提升新区承载能力。

加强旧城改造。按照“最大力度、最小动静、最好效果”的要求，狠抓老城区基础设施提升改造及城市整理，加快实施沿江综合整治提升、湖滨大道及磁湖绿道改造等工程，推进广州路及苏州路西延、黄思湾隧道复线等城市主次干道及背街小巷改造。开工各类棚改项目22个、1.6万户，实施一批老旧小区微改造整理项目，开展城区老旧住宅加装电梯试点。持续抓好城区“三难”治理，在中心城区商业繁华地段增设一批立体停车场。开工建设一批滨水景观绿化工程、山体郊野公园以及城市休憩驿站。加强违法建筑管控，新建改建一批环卫设施。加快韧性城市和智慧小区建设。

加速市域融合。启动新一轮城市总体规划修编，以大冶湖生态新区为核心，统筹谋划大冶、阳新、市开发区产业布局，健全市域交通公交化运营机制，推动中心城区与大冶、阳新城区同城化发展。加快推进以人为核心的新型城镇化，完善县城、中心镇、中心村功能，全面放开户籍门槛，实行进城人员与市民同等待遇。

（七）着力抓好以重点领域为突破口的全面深化改革

深化行政领域改革。完善智慧政务服务体系，推行“一网覆盖、一次办好”。深化商事制度改革，加快推进“多证合一”、“证照分离”，扩大“先建后验”试点范围，打造“放管服”改革升级版。大力开展“减证便民”、“证照清理”行动，让信息多跑路、群众少跑腿。深入清理整顿“红顶中介”，加快推进认证机构与政府部门彻底脱钩。推进市场管理综合行政执法改革，实现“双随机、一公开”（33）监管全覆盖。深化统计管理体制改革。分类推进事业单位改革。

加快经济领域改革。积极推进以“破、立、降”（34）为重点的供给侧结构性改革，推进“僵尸企业”市场出清，大力培育发展新动能，继续清理涉企收费，降低实体经济成本，激发各类市场主体活力。加快要素配置市场化改革，构建科技、金融、人才、土地、环保等要素资源保障体系，提高全要素生产率。完善产权制度，加快市级投融资平台市场化改革，扩大产业基金规模，提高使用效率。继续深化国有企业“三供一业”改革。

推进社会领域改革。突出抓好公立医院、药品集中带量采购、医保支付方式改革。深化教育管理体制、办学机制及考试招生制度改革。加快城市管理、文化综合执法、社会组织管理体制机制改革。完善社会信用体系，严格落实守信联合激励、失信联合惩戒制度。

（八）着力打好以抓重点、补短板、强弱项为主攻方向的“三大攻坚战”

坚决防范化解重大风险。坚持分类施策，消化存量债务，规范举债行为，优化债务结构，依法打击非法金融活动，坚决守住不发生系统性金融风险的底线。加强房地产市场监管，合理引导市场预期，坚决防止出现烂尾楼盘问题，促进房地产市场平稳健康发展。坚决防范重大安全事故、重大群体性事件、重大刑事案件发生。

全力抓好精准扶贫精准脱贫。实施产业扶贫规划，大力发展富民产业。按照搬得出、稳得住、不返贫的要求，扎实做好易地扶贫搬迁。统筹抓好农村孤儿等重点贫困对象的政策兜底工作，实施覆盖全学段的精准助学，决不让一个孩子因贫失学。完善农村贫困人口医疗保险、困难救助、定点就医先诊疗后付费等制度，决不让一个贫困户看不起病、住不了院。深入开展扶贫领域不正之风和腐败问题专项整治。加强驻村工作队建设，广泛动员社会力量参与扶贫。

坚决向污染宣战。全面抓好中央环保督察反馈问题整改销号。严格落实河长责任制，深入开展沿江环境专项整治行动，改善大冶湖等重点湖泊水质，整治彭家堑港等8个黑臭水体，新建和改造城区二、三级雨污水管网60公里。加快应急水源工程建设，推进富河流域综合治理和生态修复工程。加大保安湖、网湖湿地治理保护力度。积极创建国土资源节约集约示范市，加快实施13个土壤污染防治项目，打造国家绿色矿业发展示范区。坚决打好蓝天保卫战，持续开展“散乱污”企业、渣土车等专项整治。率先在机关事业单位开展垃圾分类试点，全面实现老城区生活垃圾减量化、无害化、资源化处理。实施宜林荒山灭荒三年行动计划，完成造林绿化8.5万亩以上，成功创建国家森林城市。建立生态保护补偿机制，扎实开展国家生态文明先行示范区建设。

（九）着力抓好以人民为中心的改善民生工作

优先发展教育事业。实施学前教育行动计划，落实新建小区配建幼儿园制度，扩大公办和普惠性民办幼儿园覆盖面。加快推进义务教育均衡发展，实施墨斗山小学、中山小学、磁湖北岸学校、黄石三中等一批中小学校新建改扩建项目，优化调整基础教育布局。对接“新高考”，实施高中阶段教育攻坚计划，提高教育教学质量。深入推进职业教育改革，加快产教融合、校企合作，推广现代学徒制试点，新增校企共建实训基地3个。支持湖北师范大学、湖北理工学院创新发展、形成特色，建设省一流学科、一流大学。鼓励社会力量办学，支持和规范民办教育发展。

加强就业和社会保障。深入实施创业引领工程、农民工返乡创业以及“我选湖北·留在黄石”大学生创业就业计划，着力化解就业结构性矛盾，努力实现更高质量、更加充分的就业。进一步完善城镇职工、城乡居民和机关事业单位养老保险制度，适时划转部分国有资本充实社保基金。及时精准实施社会救助，提高基本民生兜底保障水平。开工建设残疾人康复中心，关心关爱老年人、未成年人及妇女等特殊群体，确保弱有所助、弱有所扶。

加快健康黄石建设。完善城乡居民基本医疗保险制度，大力推行分级诊疗模式，加强医共（联）体和全科医生队伍建设，持续推动优质医疗资源向基层下沉。健全现代医院管理制度，支持鄂东医疗、鄂东医养集团持续稳定发展，支持社会力量兴办医疗、养老机构，争创全国社区和居家养老试验区。提高计生卫生服务水平，深入实施全面两孩政策，优化出生人口性别比。坚持中西医并重，积极传承发展中医药事业。实施食品药品安全放心工程。大力开展爱国卫生运动，进一步巩固创卫成果。

提升公共文化服务功能。实施文化惠民工程，加强基层“四馆三场”（35）和综合文化服务中心建设，健全公共文化服务体系。传承和保护非物质文化遗产，支持文艺精品创作，举办黄石首届艺术节，打造传统特色文化品牌。推动文化事业与文化产业互动发展，积极培育文化创意、动漫游戏、数字出版、网络视听等产业，深入推进文艺院团改革。大力创建全国文明城市，推动全民阅读，不断提高公民素质和社会文明程度。

全力办好省运会。建成省运会比赛场馆及配套工程，围绕“保三争二”目标做好备训参赛工作，确保举办一届安全、精彩、绿色、惠民的体育盛会。以筹办省运会为契机，支持鼓励城区老旧闲置厂房改造成市民健身运动场馆，在城市社区、城乡中心镇、中心村建设一批小型健身广场，方便群众体育运动。支持各类市场主体引入高水平体育赛事，大力发展运动休闲产业。

全力维护社会稳定。加快社会治安防控体系建设，实施“雪亮工程”（36），完善“九张网”功能，加快公共法律服务向基层延伸，全面提高平安产品供给率。加强网络建设和管理，打造清朗安全的网络环境。加强基层社会治理，做好村（社区）“两委”换届工作。深入推进信访制度改革，积极主动回应群众关切，依法解决群众合理诉求。全面开展安全生产领域“打非治违”（37）专项行动，深入实施公路生命安全防护工程。提升防灾减灾及应急救援能力。做好民族宗教工作。继续支持军队改革，抓好国防动员和全民国防教育，推进全国“双拥”模范城创建。

三、加强政府自身建设

新时代标注新方位，新征程承载新使命。我们将把全市人民对美好生活的向往作为奋斗目标，以永不懈怠的精神状态和一往无前的奋斗姿态，努力建设人民满意的服务型政府。

坚定政治站位。坚持用习近平新时代中国特色社会主义思想武装头脑、指导实践、推动工作，坚决维护以习近平同志为核心的党中央权威和集中统一领导，始终在思想上政治上行动上同以习近平同志为核心的党中央保持高度一致。坚持党的领导，牢固树立“四个意识”，确保中央大政方针和省市委决策部署不折不扣落地生根。严格履行重大事项向市委报告制度，自觉在市委的领导下开展工作。

坚守使命担当。按照中央和省市委的统一部署，扎实开展政府系统“不忘初心、牢记使命”主题教育。坚持勇担当善作为，深入解决经济社会发展中的重点、难点、痛点和盲点问题。坚持“大事在手、难事不躲、琐事统筹、事事着落”，继续聚焦全市重点工作、重点项目，加强督查问责，狠抓工作落实。健全激励和容错机制，为干事者鼓劲，为担当者担当。坚持务实重行，当好“施工队长”，持续“钉钉子”，抓落实、求实效、成大事。

坚持依法行政。认真贯彻法治政府建设实施纲要，完善政府法律顾问制度，推进行政决策科学化、民主化、法治化。带头学法守法，公正文明执法，全面落实行政机关负责人出庭应诉制度，提高法治素养与依法行政能力。依法接受市人大及其常委会的法律监督和工作监督，重大决策出台前主动向人大报告，自觉接受人民政协的民主监督，积极听取民主党派、工商联、无党派人士和各人民团体的意见和建议，认真办理人大代表建议和政协委员提案。完善政府信息公开、新闻发布制度，让权力在阳光下运行，切实提高政府公信力。

坚持干净干事。严格履行全面从严治党主体责任和“一岗双责”，深入推进政府系统党风廉政建设和反腐败斗争。坚持把纪律规矩挺在前面，认真贯彻落实中央八项规定实施细则及省市委实施办法，对照形式主义、官僚主义“十种表现”，扎实抓好“四风”突出问题整治。着力构建亲清新型政商关系，切实加强工程建设、土地出让、政府采购、产权交易等重点领域监管，严肃查处各类违法违纪行为和发生在群众身边的腐败问题，以建设清廉政府的实际成效取信于民。

各位代表，加速转型、赶超发展，是我们坚定前行的方向；复兴黄石、重铸辉煌，是我们矢志追寻的梦想。让我们更加紧密团结在以习近平同志为核心的党中央周围，坚持以习近平新时代中国特色社会主义思想为指导，在中共黄石市委的正确领导下，不忘初心、牢记使命，凝心聚力、砥砺奋进，让复兴黄石的时代凯歌响彻东楚，全面开启建设全省重要区域性增长极、社会主义现代化强市的新征程！
